# A missense mutation in the nuclear localization signal sequence of CERKL (p.R106S) causes autosomal recessive retinal degeneration

**Published:** 2008-10-30

**Authors:** Manir Ali, Vedam Lakshmi Ramprasad, Nagasamy Soumittra, Moin D. Mohamed, Hussain Jafri, Yasmin Rashid, Michael Danciger, Martin McKibbin, Govindasamy Kumaramanickavel, Chris F. Inglehearn

**Affiliations:** 1 Section of Ophthalmology and Neuroscience, Leeds Institute of Molecular Medicine, St. James’s University Hospital, Leeds, United Kingdom; 2 SNONGC Department of Genetics and Molecular Biology, Vision Research Foundation, Sankara Nethralaya Chennai, India; 3 Department of Ophthalmology, St. Thomas’ Hospital, London, United Kingdom; 4 Gene Tech Lab 146/1, Shadman Jail Road, Lahore, Pakistan; 5 Department of Obstetrics and Gynaecology, King Edward Medical University, Lahore, Pakistan; 6 Department of Biology, Loyola Marymount University, Los Angeles, CA; 7 Eye Department, Chancellor Wing, St. James’s University Hospital, Leeds, United Kingdom

## Abstract

**Purpose:**

To investigate the genetic basis of autosomal recessive retinal degeneration in a large consanguineous family from Pakistan.

**Methods:**

Ophthalmic examinations were conducted on family members to establish their diagnosis. Genomic DNA extracted from peripheral blood was used for homozygosity mapping to discover the chromosomal region that harbors the defective gene. Direct sequence analysis and restriction enzyme digestion were used to identify and confirm the defect in the gene.

**Results:**

There were three affected siblings in the family, each with limited peripheral vision and impaired visual acuity. We established linkage to a region on chromosome 2 that encompasses the RP26 locus. Upon sequencing the ceramide kinase-like (*CERKL)* gene, which is mutated in the original RP26 family, we identified a C>A transversion in exon 2 (c.316C>A) that substitutes an arginine residue with a serine (p.R106S) in the conserved nuclear localization signal sequence (KLKRR) of the protein. This mutation segregated with retinal degeneration in the Pakistani family and was not observed in the DNA of 174 ethnically matched unaffected controls.

**Conclusions:**

This is the third reported mutation in *CERKL* causing retinal degeneration but is the first report to show that a single amino acid change in *CERKL*, rather than a null mutation, can cause retinal disease. Although the function of CERKL is still unknown, the mutation described herein confirms that the nuclear localization signal sequence is important in the physiologic function of the protein.

## Introduction

Retinal degenerations are a heterogeneous group of eye disorders (see RetNet), which together cause a significant proportion of human blindness. A common feature is photoreceptor cell death, with conditions such as retinitis pigmentosa predominantly affecting the rod while cone and macular dystrophies lead to cone degeneration. Sometimes both rods and cones are involved, with the degeneration of one preceding the other. The patient’s symptoms can often highlight which photoreceptors are involved—for example, rod degeneration presents as night blindness or visual field constriction, while defective color vision, photophobia, or reduced visual acuity signifies a deficiency in cone function. Often a measurement of electroretinogram responses to different light intensities or colors can also assist in evaluating the primary cellular defect [1,2].

Many of the disorders that lead to retinal degeneration have a genetic basis and can be inherited in an autosomal-recessive, autosomal-dominant, or X-linked, as well as mitochondrial and polygenic manner [3-5]. There is also considerable genetic heterogeneity; for example, mutations in 21 genes have thus far been identified as causing autosomal recessive retinitis pigmentosa (RetNet). Recent efforts have focused on designing assays to assist in the rapid genetic diagnosis of retinal disease in affected individuals, as well as carriers, so as to provide genetic counseling for the patients and their families [6-8].

Here we describe a consanguineous family from Pakistan with 3 siblings who have retinal degeneration. A genome-wide screen for homozygosity was performed, and a region at the RP26 locus [9] was found to cosegregate with the disease phenotype. A novel mutation in the ceramide kinase-like (*CERKL)* gene was identified. This is the third report of a mutation in the *CERKL* gene causing autosomal recessive retinal degeneration.

## Methods

### Patients and controls

**Figure 1:**
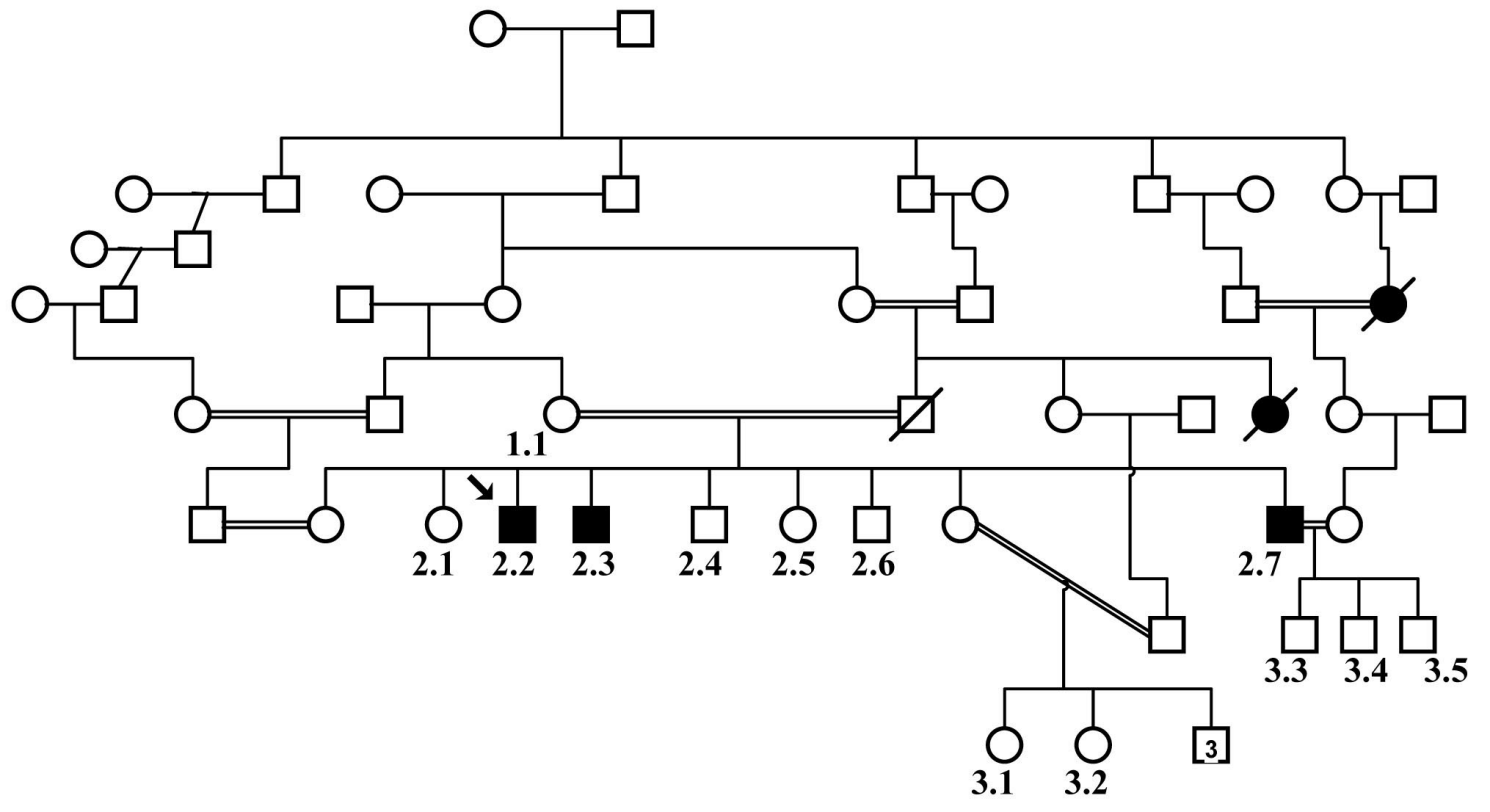
Pedigree structure. Pedigree of the Pakistani family shows affected members (shaded) who have retinal degeneration and those individuals who are unaffected (unshaded). The arrow marks the proband. The numbers mark the family members from whom DNA is available.

The study of human subjects was performed according to the principles of the Declaration of Helsinki using a process approved by a UK ethics committee. The proband was one of three affected siblings with deteriorating vision who were part of a large consanguineous family from Lahore in Pakistan (see Figure 1). After obtaining informed consent from the elder of each household, we conducted an ophthalmic examination and took a sample of peripheral blood from the family members. Genomic DNA was extracted from the blood using the QIAamp DNA Blood Midi Kit (Qiagen, Crawley, UK) according to the manufacturer’s instructions. Control subjects were unrelated normal individuals who were recruited as siblings of patients subject to genetic testing by the Yorkshire Regional Genetics screening service at St. James’s Hospital, Leeds. None of the families involved had any member with an inherited eye abnormality and all the individuals were of Asian subcontinent extraction.

### Homozygosity mapping

Aliquots of DNA from affected and unaffected family members were genotyped for over 400 markers covering all human chromosomes by the Marshfield Institute. Candidate homozygous regions were further analyzed with additional markers that intersected the Marshfield data set using fluorescently labeled primers. The products were mixed with the size standard GeneScan 500-ROX (Applied Biosystems, Warrington, UK) and resolved by electrophoresis on a 3130xl Genetic Analyzer (Applied Biosystems). The results were analyzed using the GeneMapper version 4.0 software (Applied Biosystems). Pedigree and haplotype data were managed with the Cyrillic package version 2.1. A multipoint linkage analysis was performed using the LinkMap from the Linkage suite of programs [10].

### DNA sequencing

Specific primer pairs encompassing the 14 coding exons, as well as the intron-exon boundaries, of the *CERKL* gene have been described before [11]. These were used (Table 1) in the PCR to amplify products that were initially digested with ExoSAP-IT (GE Healthcare, Chalfont St. Giles, UK) according to the supplier’s instructions. The digested DNA was sequenced directly using the BigDye Terminator version 3.1 Cycle Sequencing Kit and the 3130xl Genetic Analyzer according to the manufacturer’s instructions (Applied Biosystems).

**Table 1:** Oligonucleotide primer pairs used for the amplification of *CERKL* exons.

**Exon**	**Primer sequence (F)**	**Primer sequence (R)**	**Product size (bp)**
1	dACTTTACAGGAGAAGCTAGGCG	dCTTGTGACGTTTGCCCGG	787
2	dCCATCTCACGTAGGGACTGG	dTATACACCATGGCCATTGGG	900
3	dCTAACAGACTTGTGTGTC	dCCCAAGTTTGCATTAAGGAC	305
4	dGCCAGAACAAGTTAAAAAGTGTG	dTGTAGCTAAAACTAGTGAAGGCA	209
4a	dGTCTGTGATAGATTGAGGGAAGA	dCACATCAGTCCAACACTTTAGC	280
5	dGGTACATGTGAGCAGTTATGCAC	dTAGTGGGGATGCCAGAAGTC	399
6	dCATCTGAACATTGAAGAATGAC	dGAGACAAAGAACCTGCCTTT	283
7+8	dGCTCTCTTATGTTTGCTG	dCTGATCAATTGTTTGTCAGAATG	460
9+10	dCCTACTGTGATGACAAATCCC	dGGCAGCAACAAAATTGTACG	548
11	dCATGGTGATTTATCTATCTTGTCCA	dCAATTCTTGCAGCATCTTTTTC	299
12	dCTTGTGAGAGAGGGCTCAGTG	dCCAACTGCCTGCTTTGATAGTTC	358
13	dGGCATTGGCATTGTGTACC	dCTGAGGTGGAACAGTTCATCC	308

The expected size of each amplified product is depicted.

### Mutation restriction analysis

To screen for the c.316C>A mutation in additional family members and control DNAs, we performed PCR. We employed a forward primer that had been designed with a deliberate mismatch at the fourth residue from the 3-prime end (underlined; C instead of an A nucleotide: dAAA GAC ATA TTC TCT GTG AAA CTG AAC CGG) and a reverse primer (dCCA TAT GTC ACA GTG GTC TTC), and we used an annealing temperature of 58 °C. The PCR product containing the wildtype and/or mutant sequence was digested with the restriction endonuclease BsaWI (W↓CCGGW) (New England Biolabs, Hitchen, UK). After incubation at 60 °C, the reaction products were resolved through a 2% agarose gel by electrophoresis. For wildtype sequence, a single band of 168 bp would be expected, however for a mutant sequence the product would be digested to yield 140 and 28 bp.

### Bioinformatics

Comparative genomics for protein sequence conservation across mammalian and non-mammalian vertebrates were investigated using the University of California Santa Cruz Genome Browser, which is maintained by the Genome Bioinformatics Group there. This study used the Vertebrate Multiz Alignment and PhastCons Conservation package.

## Results

We evaluated autosomal recessive retinal degeneration in a Pakistani family that included three brothers with poor vision. The parents are first cousins and are unaffected, suggesting that the visual deficit is due to a mutation transmitted from a common ancestor as an autosomal recessive trait. The three affected brothers were examined at 29, 36, and 42 years of age. All reported nyctalopia and loss of visual field from early in the second decade, followed by loss of visual acuity, often with photophobia, from the end of the second or early in the third decade. At the time of examination, acuity was limited to perception of light only in two brothers and no perception of light in one brother. Two brothers had roving eye movements. Mean refractive error was −3.8 diopters. The eyes of the affected individuals, all had posterior subcapsular cataract, except for one which was pseudophakic. Disc pallor and arterial attenuation were universal findings. Bone spicule pigmentation was seen anterior and posterior to the equator in all eyes, with white dots in the outer retina anterior to the temporal vascular arcades. Despite the visual acuity, the only macular abnormality was cellophane maculopathy in 5 of 6 eyes. As the patients were seen in Pakistan, it was not possible to obtain electroretinograms or fundus photographs.

**Figure 2:**
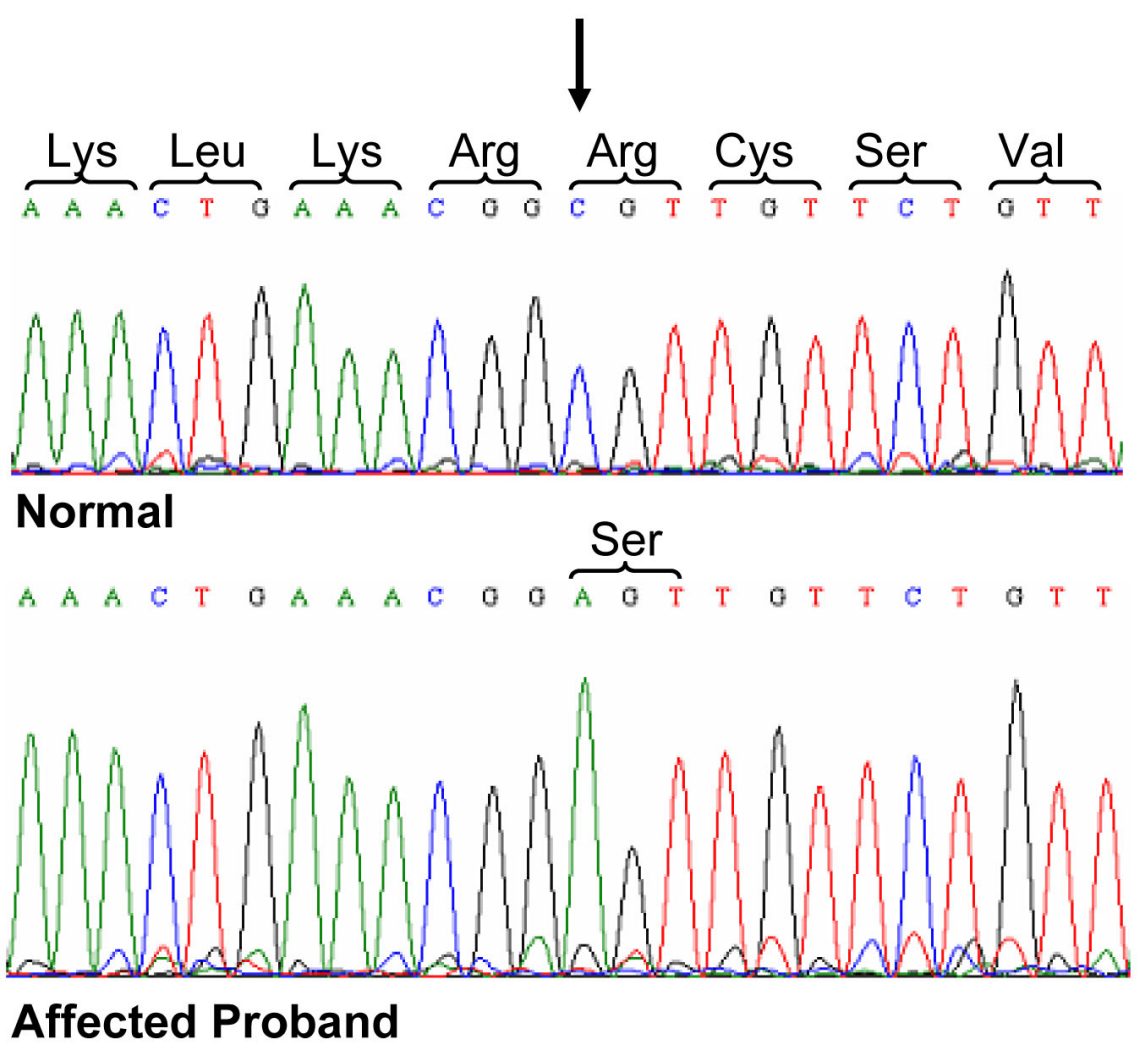
Sequence analysis of the *CERKL* gene. The sequencing chromatograms show the forward strand of *CERKL* exon 2 from a normal control and the affected proband. Depicted is a single nucleotide change C>A (shown by the arrow) in the DNA of the affected proband, which creates a missense change in the protein sequence replacing an arginine (Arg) with a serine (Ser) residue.

**Figure 3:**
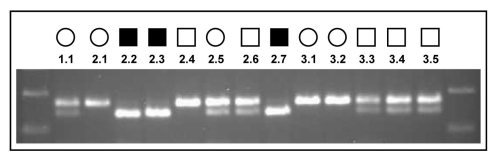
Analysis of the c.316C>A mutation. Ultraviolet illuminated agarose gel presents BsaWI-digested PCR products from the DNA of each of the family members. A band size of 168 bp represents an uncut digest that would exist in the presence of a wildtype sequence. The presence of a digested 140 bp band occurs when a mutant sequence is present. The 28 bp band following digestion of the mutant sequence cannot be seen as it has run off the gel. Note the segregation of the mutation with the disease phenotype so that only the DNA from the affected individuals gives rise to a single 140 bp band after digestion of the PCR.

To identify the defective gene causing retinal degeneration in this family, we sent genomic DNA from affected individuals and their relatives to the Marshfield Institute Mammalian Genotyping Service, Marshfield, WI for homozygosity mapping. This highlighted a region of homozygosity on chromosome 2 at the RP26 locus, previously implicated in recessive RP. We therefore screened the DNA of one of the affected brothers (the proband) and a normal control individual for the 14 coding exons and splice site junctions of *CERKL*, the gene implicated in the RP26 form of inherited blindness. Following direct sequence analysis, we identified a C>A transversion in exon 2 (c.316C>A) that substitutes an arginine residue with a serine (p.R106S) in the protein sequence (Figure 2). This was the only sequence variant identified during the analysis. This single base change segregates with the disease phenotype (Figure 1 and Figure 3) and was excluded from the DNA of 174 ethnically matched unaffected individuals.

**Figure 4:**
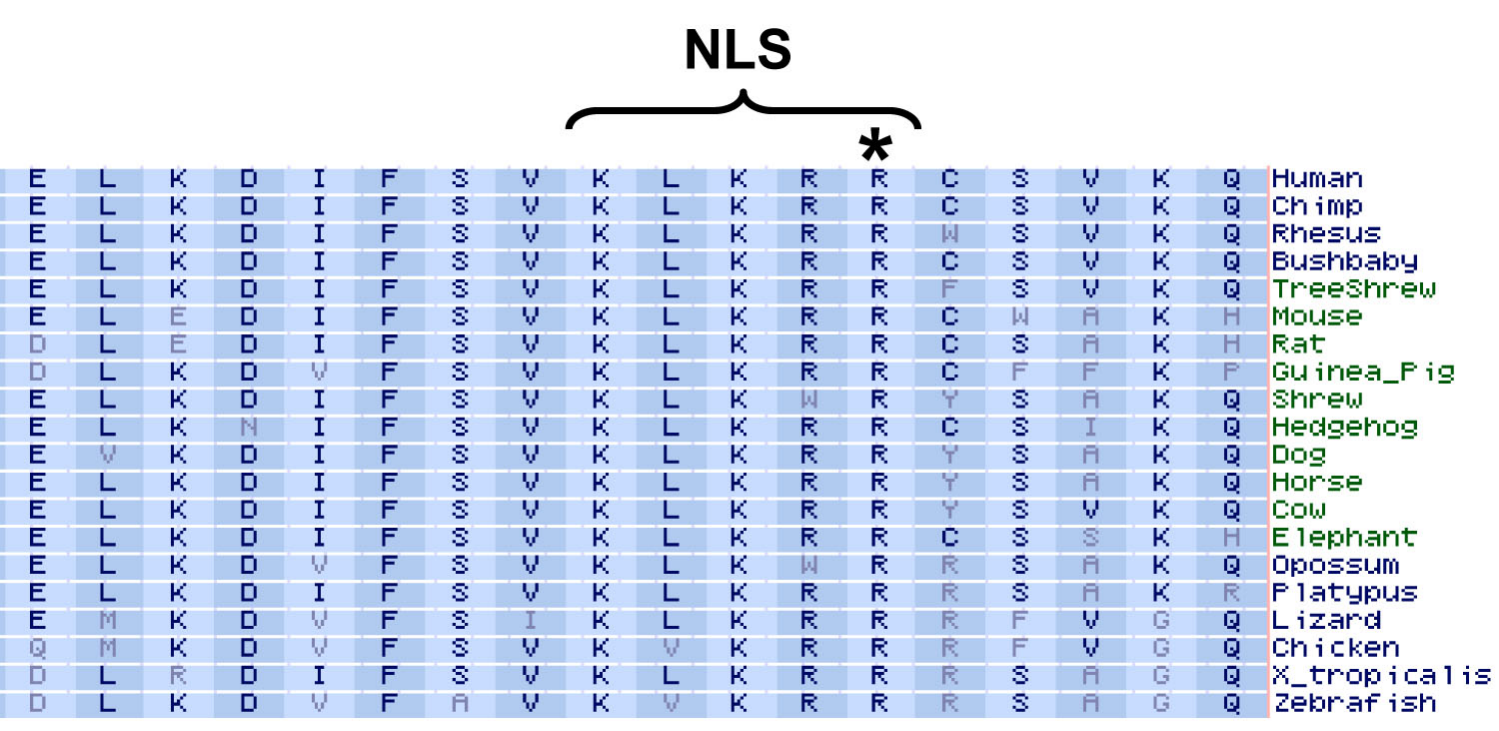
Protein sequence conservation. Diagram shows part of the amino acid sequence of the CERKL protein from multiple species around the nuclear localization signal sequence (NLS), which is represented as “KLKRR” in humans. Note the asterisk (*), which marks the evolutionary conserved arginine (R) residue in the normal sequence that is mutated to a serine in patients who go on to develop retinal degeneration.

The arginine at position 156 in CERKL is highly conserved among other species (Figure 4). The mutation is likely to disrupt the nuclear localization signal sequence “KLKRR” that is necessary for targeting CERKL to the nucleus and for enriching the protein in the nucleolus [12]. A serine residue at position 156 is therefore highly likely to be pathogenic and would be expected to account for the retinal degeneration seen in this family.

## Discussion

In this paper we report the identification of a novel mutation in the *CERKL* gene (c.316C>A, p.R106S) that causes retinal degeneration in a large consanguineous family from Pakistan with 3 affected siblings. This is the third reported case of *CERKL* gene mutations causing retinal disease and the first presenting a missense mutation, rather than a null allele. Previous reports have identified a premature stop codon mutation as the cause of disease in the original RP26 family from Spain as well as several other families of Spanish origin (p.R257X) [13,14]. Auslender et al. reported a mutation in a splice site signal that destroyed the splice recognition sequence as a founder mutation that caused retinal disease in a Yeminite Jewish population (c.238+1G>A) [11]. Examinations performed on the affected individuals from these three studies as well as this study have highlighted similar clinical features, involving both rod and cone degeneration, the presence of retinal pigmentary deposits and macular alterations leading to a deficit in both peripheral and central vision.

The function of CERKL in the human retina is unknown. CERKL transcripts have been shown to be expressed in the ganglion and photoreceptor layers of the retina [13], and generally show a restrictive pattern of expression in tissues, compared to the homolog, CERK, which is ubiquitously expressed [13,15]. CERKL has a kinase motif as well as a putative Ca^2+^/calmodulin binding domain at the C-terminal end [15]. However, the activity of CERKL is still unknown, unlike CERK, which phosphorylates ceramide to ceramide-1-phosphate [16]. Nevertheless, clues to CERKL regulation and function come from the observation that it can be phosphorylated [15] and also that it localizes to the nucleolus [12,15]. The former requires further investigation but the latter has been investigated. Subcellular localization studies following overexpression of tagged CERKL into transfected cells have highlighted its distribution in the cytoplasm, nucleus, and in particular its enrichment in the nucleolus (15). CERKL possesses a nuclear localization signal sequence (102-KLKRR-106) that permits the active transport of CERKL from the cytoplasm into the nucleus [12]. A single amino acid substitution, which disrupts the nuclear localization signal (p.R105A), prevents the mutant CERKL from localizing to the nucleus [12], as does the splice site variant, del1–205, which lacks the nuclear localization sequence altogether [15]. Interestingly, the CERKL mutants, p.R257X, p.R379X, and p.G234D, which contain the nuclear localization sequence, are indeed transported into the nucleus but do not localize to the nucleolus, suggesting that other features of CERKL contribute to the nucleolar localization [15]. Treatment with a calcium ionophore also prevents localization of wildtype CERKL to the nucleolus [15]. It is noteworthy that the missense mutation identified in the Pakistani family reported in this paper (p.R106S) would be expected to disrupt the nuclear localization signal sequence. Thus the mechanism for triggering retinal degeneration in these patients could be the absence of nuclear localization and subsequent accumulation of mutant CERKL in the cytoplasm, which might be expected to trigger apoptotic cell death.

The wildtype proteins corresponding to other ‘retinal degeneration’ genes have also been shown to localize to the nucleolus. These include the oxygen-regulated photoreceptor protein (ORP1) and tubby-like protein 1 (TULP1) [17,18], and mutations in the corresponding genes have been shown to cause RP1 and RP14 [19,20]. The nucleolus, which contains rRNA genes and the factors for ribosome and ribonucleoprotein particle biosynthesis, is a multifunctional organelle that is now thought to be involved in the regulation of mitosis, cell cycle progression and many forms of stress response [21]. CERKL may have an important role in any of these processes in the cells of the retina and the functional link between CERKL and it’s nucleolar localization warrants further study.
